# BiOCl Atomic Layers with Electrons Enriched Active Sites Exposed for Efficient Photocatalytic CO_2_ Overall Splitting

**DOI:** 10.1007/s40820-025-01723-2

**Published:** 2025-04-18

**Authors:** Ting Peng, Yiqing Wang, Chung-Li Dong, Ta Thi Thuy Nga, Binglan Wu, Yiduo Wang, Qingqing Guan, Wenjie Zhang, Shaohua Shen

**Affiliations:** 1 International Research Center for Renewable Energy, State Key Laboratory of Multiphase Flow in Power Engineering, Xi’an Jiaotong University, Xi’an, 710049 People’s Republic of China; 2 Department of Physics, Tamkang University, New Taipei City, 25137 Taiwan People’s Republic of China

**Keywords:** Photocatalysis, CO_2_ overall splitting, BiOCl atomic layers, Charge separation

## Abstract

Given the limited exposure of active sites and the retarded separation of photogenerated charge carriers in those developed photocatalysts, photocatalytic CO_2_ splitting into value-added chemicals has suffered from the poor activity and remained in great challenge for real application. Herein, hydrothermally synthesized BiOCl with layered structure (BOCNSs) was exfoliated into thickness reduced nanosheets (BOCNSs-w) and even atomic layers (BOCNSs-i) via ultrasonication in water and isopropanol, respectively. In comparison with the pristine BOCNSs, the exfoliated BiOCl, especially BOCNSs-i with atomically layered structure, exhibits much improved photocatalytic activity for CO_2_ overall splitting to produce CO and O_2_ at a stoichiometric ratio of 2:1, with CO evolution rate reaching 134.8 µmol g^−1^ h^−1^ under simulated solar light (1.7 suns). By surpassing the photocatalytic performances of the state-of-the-art Bi_l_O_m_X_n_ (X: Cl, Br, I) based photocatalysts, the CO evolution rate is further increased by 99 times, reaching 13.3 mmol g^−1^ h^−1^ under concentrated solar irradiation (34 suns). This excellent photocatalytic performance achieved over BOCNSs-i should be benefited from the shortened transfer distance and the increased built-in electric field intensity, which accelerates the migration of photogenerated charge carriers to surface. Moreover, with oxygen vacancies (V_O_) introduced into the atomic layers, BOCNSs-i is exposed with the electrons enriched Bi active sites that could transfer electrons to activate CO_2_ molecules for highly efficient and selective CO production, by lowering the energy barrier of rate-determining step (RDS), *OH + *CO_2_^−^ → HCO_3_^−^. It is also realized that the H_2_O vapor supplied during photocatalytic reaction would exchange oxygen atoms with CO_2_, which could alter the reaction pathways and further reduce the energy barrier of RDS, contributing to the dramatically improved photocatalytic performance for CO_2_ overall splitting to CO and O_2_.

**Supplementary Information:**

The online version contains supplementary material available at 10.1007/s40820-025-01723-2.

## Highlights

- BiOCl atomic layers (BOCNSs-i) were prepared by exfoliating hydrothermally synthesized BiOCl (BOCNSs) via ultrasonication in isopropanol for efficient photocatalytic CO_2_ overall splitting to CO and O_2_.
- The obtained BOCNSs-i photocatalyst exhibits a distinctly improved photocatalytic performance to stoichiometrically produce CO and O_2_ at the ratio of 2:1, with the CO evolution rate reaching 134.8 µmol g^−1^ h^−1^ under simulated solar light (1.7 suns) and reaching 13.3 mmol g^−1^ h^−1^ under concentrated solar irradiation (34 suns).
- With the thickness of BiOCl photocatalyst reducing to atomic layers, the charge carrier transfer and separation were enhanced by shortened transfer distance and the increased built-in electric field intensity, and electrons enriched Bi sites were exposed for activating CO_2_ molecules.

**Supplementary Information:**

The online version contains supplementary material available at 10.1007/s40820-025-01723-2.

## Graphical abstract

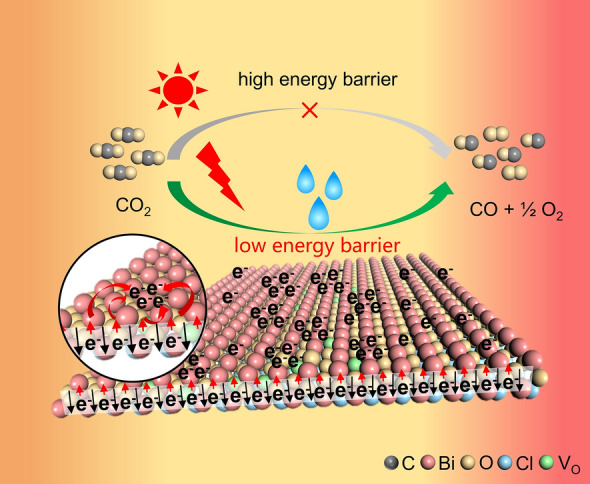

## Introduction

Solar-driven photocatalysis converting CO_2_ to fuels is a promising strategy to cope with greenhouse effect and energy dilemma [1–3]. However, the low-density exposure of reactive sites and the fast recombination of photogenerated electrons and holes in those developed photocatalysts significantly inhibit the photocatalytic CO_2_ reduction activity and thus impose the inevitable restriction on its practical implementation [4, 5]. With thickness reduced to be several nanometers and even atomic layers, two-dimensional (2D) ultrathin architectures acting as photocatalysts are believed to reduce charge carrier recombination on account of the shortened diffusion distance from bulk to surface. In addition, 2D ultrathin photocatalysts possess much increased specific surface areas that expose abundant reactive sites for sufficient atom utilization and thus much improved photocatalytic activities [5–8].

As a kind of well-studied 2D semiconductors for photocatalysis, bismuth oxyhalides, Bi_l_O_m_X_n_ (X: Cl, Br, I), are featured with layered structure constructed by [Bi_l_O_m_] and [X_n_] layers. Given the strongly covalent-bonded atoms in monolayers and the week van der Waals force between monolayers, Bi_l_O_m_X_n_ is hospitable to be exfoliated to ultrathin nanosheets, endowed with anisotropic carrier transfer property, and thus promoted charge separation ability [9–11]. For example, in comparison with bulk Bi_3_O_4_Br, the Bi_3_O_4_Br nanosheets with ultrathin thickness of about 1.7 nm possessed a remarkably improved charge separation efficiency, owing to the shortened diffusion distance of photogenerated carriers from bulk to surface [12]. Interestingly, with thickness reduced from 120 to 30 nm by liquid-phase exfoliation, the built-in electric field was strengthened significantly in the obtained Bi_3_O_4_Cl single-crystal nanosheets, contributing to the much enhanced photogenerated carrier separation and transfer ability [13]. It should be also noted that for 2D structures with dimension reduced to atomic layers, the specific areas are bound to be dramatically increased with plentiful active sites exposed for photocatalytic reactions. Guan et al. hydrothermally prepared atomically layered BiOCl nanosheets (~ 2.7 nm) with O atoms escaped from the [Bi_2_O_2_] layers and then triple V_Bi_^‴^V_O_^••^V_Bi_^‴^ vacancy associates formed at surface; and the resulted synergic advantages of enhanced optical adsorption capability, effective charge carrier separation, and more reductive photoexcited electrons contributed to the significant promotion in photocatalytic performance [14].

Given the tunable layered structure of Bi_l_O_m_X_n_ (X: Cl, Br, I), liquid-phase ultrasonication has been evidenced effective to break the week van der Waals force between monolayers to obtain ultrathin or atomically layered Bi_l_O_m_X_n_ nanosheets, with surface oxygen atoms escaped from lattice and then oxygen vacancies created at surface under harsh energy input [9, 15]. Indeed, oxygen vacancies would act as active centers or regulate the electronic structures of active sites, thus promoting the photocatalytic performances [4]. For instance, by ultrasonically treating pristine BiOCl nanosheets in toluene, Chen et al. obtained BiOCl atomic layers, decorated with oxygen vacancies acting as active sites for the promoted activation of CO_2_ molecules, which achieved a CO evolution rate of 8.99 μmol g^−1^ h^−1^, 3.8 times that of bulk BiOCl [15]. For the ultrasonication exfoliated BiOBr atomic layers with abundant oxygen vacancies created at surface by UV irradiation, the altered charge density distribution around oxygen vacancies favored CO_2_ adsorption, and an improved photocatalytic activity was realized for CO_2_ reduction, with CO production rate reaching 87.4 μmol g^−1^ h^−1^, 24 times higher than that of bulk BiOBr [16].

As inspired the above demonstrations, in this study, hydrothermally synthesized BiOCl with layered structure (BOCNSs) was ultrasonicated in water and isopropanol, respectively, and exfoliated into thickness reduced nanosheets (BOCNSs-w) and even atomic layers (BOCNSs-i) as photocatalysts for highly efficient and selective CO production via CO_2_ reduction. In comparison with the pristine BOCNSs (thickness: 8–9 nm), both BOCNSs-w (thickness: 4–5 nm) and BOCNSs-i (thickness: 2–3 nm) with thickness significantly reduced exhibit extremely improved photocatalytic performances for CO_2_ overall splitting, with CO and O_2_ simultaneously produced at a stoichiometric ratio of 2:1. Especially for BOCNSs-i, the CO evolution rate reaches 134.8 µmol g^−1^ h^−1^ under simulated solar light (1.7 suns), 11.0 and 7.3 times that of BOCNSs and BOCNSs-w, respectively. Encouragingly, the CO evolution rate could be increased further by ~ 99 times, reaching 13.3 mmol g^−1^ h^−1^ as high for BOCNSs-i under concentrated solar irradiation (34 suns). This atomically layered BOCNSs-i is believed to be benefited from the shortened carrier migration distance and the promoted charge carrier separation and transfer to the surface. Moreover, with oxygen vacancies (V_O_) introduced into the atomic layers, the electrons enriched Bi active sites are well exposed for highly efficient and selective CO production. In situ spectral investigations and theoretical calculations reveal that the electrons enriched Bi active sites would transfer electrons to activate CO_2_ molecules, reducing the energy barrier of RDS, i.e., *OH + *CO_2_^−^ → HCO_3_^−^. Isotope label experiments confirm that H_2_O supplied during photocatalytic reaction would exchange oxygen atoms with CO_2_, which further reduces the energy barrier of RDS by altering the reaction pathways and then contributes to the dramatically improved photocatalytic performance for CO_2_ overall splitting to CO and O_2_. This work provides a facile approach to the controllable fabrication of atomically layered nanostructures with active sites exposed for efficient photocatalysis and also deepens the understanding of molecular and atomic fundamentals for photocatalytic CO_2_ overall splitting.

## Experimental Section

### Materials

All the materials were used as received without further purification. Deionized water, with a resistivity of 18.25 MΩ·cm, was used throughout the experiments. Bismuth nitrate pentahydrate (Bi(NO_3_)_3_·5H_2_O, 99%) and mannitol (C_6_H_14_O_6_, 99%) were purchased from Meryer (Shanghai) Biochemical Technology Co., Ltd. Polyvinyl pyrrolidone (PVP, k30, Mw =  ~ 40,000) was purchased from Shanghai Aladdin Biochemical Technology Co., Ltd. Sodium chloride (NaCl, 99.9%) was purchased from China National Pharmaceutical Group Co., Ltd.

### Synthesis of BOCNSs

Solution A was prepared by dissolving 400.0 mg of PVP in 25 mL of 40 mmol L^−1^ mannitol solution. Solution B was prepared by dissolving 1 mmol of Bi(NO_3_)_3_·5H_2_O in 5 mL of Solution A. Transparent solution C was formed by Solution B added dropwise into Solution A. Then, 5 mL of NaCl saturated solution was dropped slowly into solution C under vigorous stirring. The resultant solution was transferred into 50 mL Teflon-lined autoclave for hydrothermal reaction for 24 h at 160 °C. The resultant sediment was dried in vacuum at 60 °C after being washed with ultrapure water and ethanol for several times. The obtained sample was denoted as BOCNSs.

### Synthesis of BOCNSs-w

Two hundred mg of BOCNSs was dispersed in 250 mL of ultrapure water and treated by ultrasonication for 4 h at 30 °C. The obtained suspension liquid was centrifuged at 11,000 r min^−1^ for 10 min. The resultant supernate was freeze-dried for 48 h. The obtained sample was donated as BOCNSs-w.

### Synthesis of BOCNSs-i

Two hundred mg of BOCNSs was dispersed in 250 mL of isopropanol and treated by ultrasonication for 4 h at 30 °C. The obtained suspension liquid was centrifuged at 11,000 r min^−1^ for 10 min. The resultant supernate was rotary evaporated at 40 °C. The moist powder was dispersed in ultrapure water and freeze-dried for 48 h. The obtained sample was donated as BOCNSs-i.

### Photocatalytic CO_2_ Overall Splitting Test

Photocatalytic CO_2_ overall splitting was conducted in a 130 mL quartz reactor, in which a piece of FTO glass (fluorine-doped SnO_2_ conductive glass, 2 × 2 cm^2^) sheets evenly covered by 5 mg of photocatalyst was placed at bottom. CO_2_ (99.999%) gas was purged into the quartz reactor after vacuumized for 5 times. Then, 200 μL of ultrapure water was injected into quartz reactor, with reaction temperature kept at 25 °C by cooling water. Photocatalytic CO_2_ overall splitting test conducted under irradiation by a 300 W Xe lamp for 4 h. The amounts of evolved CO and O_2_ were measured by a gas chromatograph (BRUKER 450-GC) equipped with capillary column and another gas chromatograph (BRUKER 450-GC) equipped with NaX zeolite column. For cycling tests, the photocatalysts after each reaction were dried at 60 °C for 12 h in a vacuum oven, which was then used for next photocatalytic test. Products of ^13^CO_2_ (99%) and H_2_^18^O isotope labeling experiments over BOCNSs-i were detected by an Agilent 8860-5977B gas chromatograph–mass spectrometer.

### Characterization

Scanning electron microscopy (SEM) images were recorded using JEOL 7800F field emission scanning electron microscope. Transmission electron microscopy (TEM) images were collected by a Thermo Fisher Scientific Talos F200X Lorenz transmission electron microscope operated at an accelerating voltage of 200 kV. The aberration-corrected high-angle annular dark-field scanning transmission electron microscopy (AC HAADF-STEM) images were recorded by a JEOL JEM-ARM-200CF transmission electron microscope equipped with single spherical aberration (Cs) correctors. Atomic force microscopy (AFM) images and surface potentials were measured on a Shimadzu SPM-9700HT atomic force microscope equipped with Kelvin probe force microscope (KPFM). Specific surface areas and CO_2_ physical adsorption isotherms were measured using Micromeritics ASAP 2020 analyzer. X-ray diffraction (XRD) patterns were obtained on a PANalytical X’Pert Pro MPD diffractometer operated at 40 kV and 40 mA equipped with Ni-filtered Cu Kα irradiation (*λ* = 1.5406 Å). Raman spectra were recorded using a Renishaw InVia Qontor laser Raman spectrometer equipped with 633 nm laser device. Electron spin resonance (ESR) spectra were collected using a Bruker EMX X-band spectrometer and microwave frequency = 9.40 GHz at room temperature. X-ray absorption near-edge structure (XANES) spectra and extended X-ray absorption fine structure (EXAFS) spectra at Bi L_3_-edge were recorded at BL20A beamline at the National Synchrotron Radiation Research Center, Taiwan. UV–Vis diffuse reflectance spectra (DRS) were carried out using an Agilent Cary 5000 UV–visible-NIR spectrophotometer. Zeta potentials were measured on a Malvern Zetasizer Nano ZS. The steady-state photoluminescence emission (PL) spectra and time-resolved transient photoluminescence (TRPL) decay spectra were collected at an Edinburgh Instruments FLS1000 fluorescence spectrophotometer at room temperature.

### Photoelectrochemical Measurements

Photoelectrochemical measurements were conducted on a Metrohm Autolab PGSTAT 302N electrochemical workstation with a three-electrode system. Platinum foil and Ag/AgCl electrode were used as counter and reference electrodes, respectively. The working electrodes were prepared by dropping a suspension liquid (1 mg of photocatalyst, 0.5 mL of ethanol, 0.5 mL of ultrapure water, and 10 μL of Nafion) on FTO glass sheets and naturally dried at room temperature. The area covered by photocatalyst on FTO glass was fixed at 1 × 1 cm^2^. 0.1 M Na_2_SO_4_ aqueous solution was used as the electrolyte. The transient photocurrent curves were measured at an applied potential of 0.2 V versus Ag/AgCl with an interval of 15 s on/off switching. Electrochemical impedance spectra were recorded at an applied potential of 0.8 V versus Ag/AgCl.

### In Situ X-Ray Photoelectron Spectroscopy (XPS) Experiments

In situ XPS experiments were performed by a Thermo Fisher Scientific ESCALAB Xi + , which was equipped with IPES vacuum system. Generally, sample was placed in in situ reactor and pretreated at 180 °C for 30 min by pure N_2_ (99.999%) at a pressure of 2.5 bar. CO_2_ was introduced to the reactor for saturated adsorption, and XPS signals were collected in dark and under illumination. Then, a mixed gas of CO_2_ and H_2_O vapor was introduced into the reactor for saturated adsorption under illumination, and XPS signals were collected. All the binding energies were calibrated by the C 1*s* peak at 284.8 eV.

### In Situ Diffuse Reflectance Infrared Fourier Transform Spectroscopy (DRIFTS) Experiments

In situ DRIFTS experiments were conducted by a Bruker INVENIO X Fourier transform spectrometer, which equipped with MCT (Mercury Cadmium Telluride) detector, Harrick BDRK-4-BR4 in situ diffusing device and IKARC2lite cooling circulating machine. Sample was placed in Harrick BDRK-4-BR4 in situ diffusing device and pretreated at 180 °C for 30 min by pure N_2_ (99.999%) with a flow rate of 10 mL min^−1^. The signal in N_2_ atmosphere was recorded as background when sample was chilled to the circumstance temperature. A mixed gas of CO_2_ and H_2_O vapor was introduced to the sample surface in dark, and signals were collected every 5 min until saturated adsorption. Then, signals were collected every 5 min under illumination.

#### In Situ Raman Spectroscopy Experiments

In situ Raman spectroscopy experiments were carried out on a Renishaw InVia Qontor laser Raman spectrometer, equipped with a 633 nm laser device. Sample was placed in a homemade in situ Raman cell, which was purged with pure Ar (99.999%) at a flow rate of 10 mL min^−1^ for 30 min before experiments. With the mixed gas of CO_2_ and H_2_O vapor introduced to the in situ cell, the signals were collected every 5 min in dark, until saturated adsorption. Then, the signals were collected every 5 min under illumination of a 300 W Xe lamp.

#### Density Functional Theory (DFT) Calculations

DFT calculations were performed using the CASTEP code, with the Bi site on the BiOCl(001) surface considered as the active site for CO₂ reduction. A 4 × 2 unit cell model was employed to simulate the lateral dimensions. To construct different oxygen vacancy models, we selected four atomic layers without oxygen vacancies, two atomic layers with 36 oxygen vacancies, and one atomic layer with 72 oxygen vacancies, corresponding to the BOCNSs, BOCNSs-w, and BOCNSs-i models, respectively. To minimize periodic interactions between cells, a 20 Å vacuum layer was added. Geometric structure optimizations were carried out using the Perdew–Burke–Ernzerhof (PBE) functional within the Generalized Gradient Approximation (GGA). A 4 × 4 × 1 Monkhorst–Pack k-point grid was used for the models. The convergence criteria for electronic structure iterations were set to 1.0 × 10^−5^ eV atom^−1^ (energy) and 0.01 eV Å^−1^ (maximum force). To more accurately describe the interaction between the reactants and the catalytic surface, the DFT-D3 dispersion correction was applied. The Gibbs free energy for each gaseous and adsorbed species was calculated at 298.15 K using the following equation:

G = E_DFT_ + E_ZPE_ − TS.

Where E_DFT_ represents the electronic energy obtained from CASTEP calculations, E_ZPE_ is the zero-point energy, T is the temperature, and S is the entropy contribution. Zero-point energy and entropy were computed using the standard ideal gas method.

## Results and Discussion

### Structure of Exfoliated BiOCl Nanosheets

**Fig. 1:**
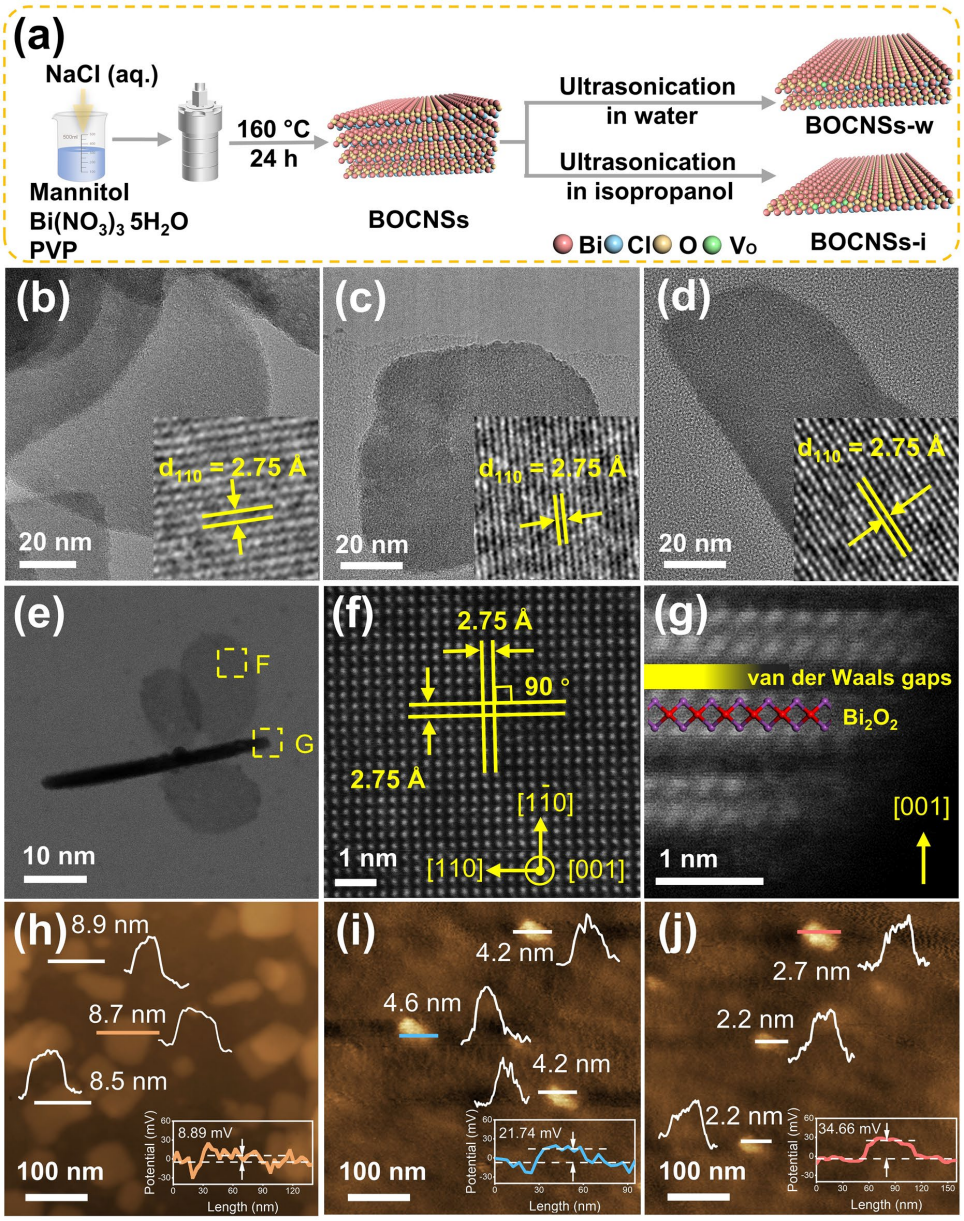
**a** Schemed fabrication of BOCNSs-w and BOCNSs-i via liquid-phase exfoliation of BOCNSs obtained by hydrothermal method. TEM and HRTEM images of **b** BOCNSs, **c** BOCNSs-w, and **d** BOCNSs-i. **e** AC HAADF-STEM image, **f** [001] facet-oriented, and **g** [110] facet-oriented TEM images of BOCNSs-i. AFM images and corresponding potential curves of **h** BOCNSs, **i** BOCNSs-w, and **j** BOCNSs-i

**Fig. 2:**
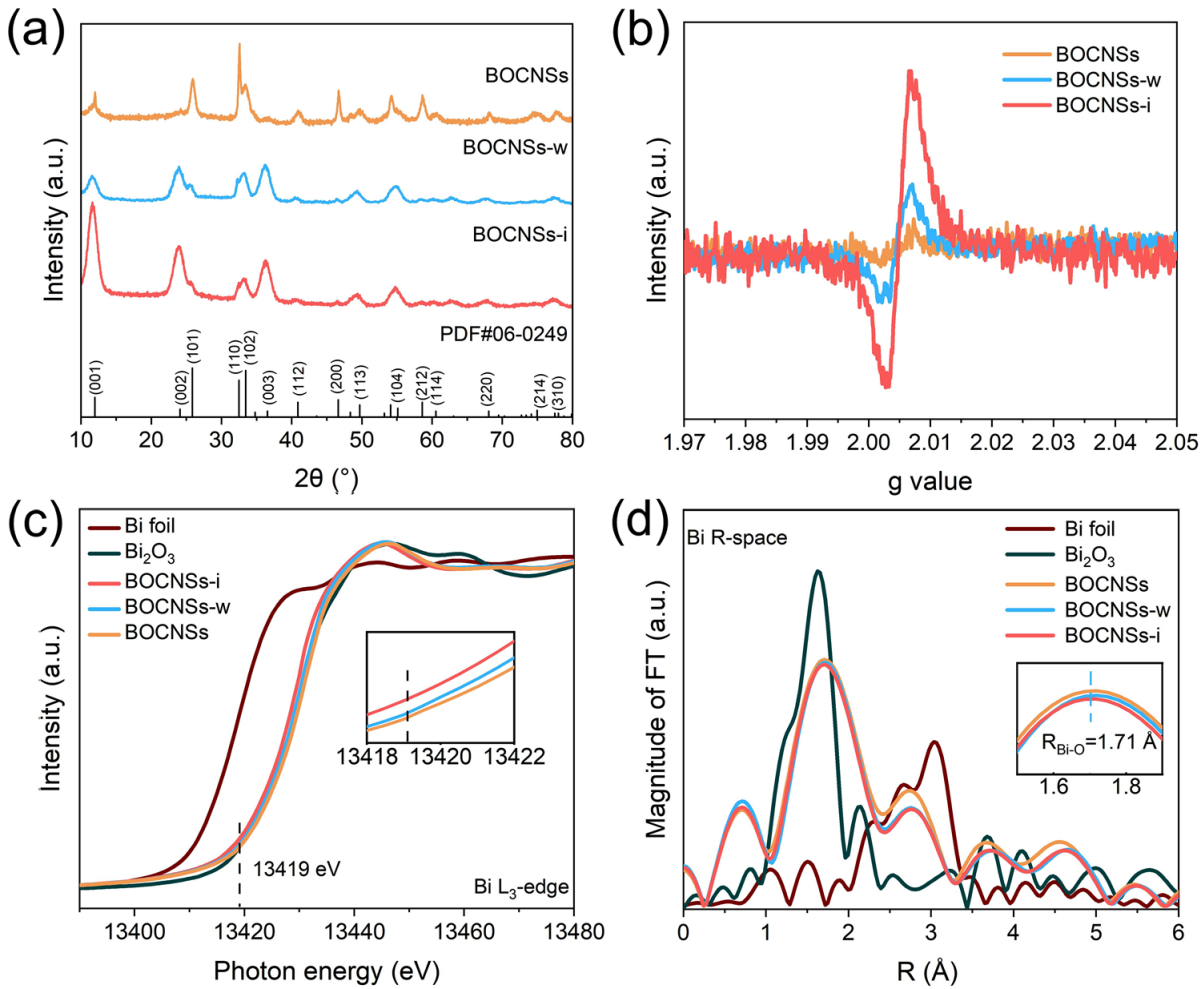
**a** XRD patterns, **b** electron spin resonance spectra, **c** Bi L_3_-edge XANES spectra, and **d** Bi R-space EXAFS spectra of BOCNSs, BOCNSs-w, and BOCNSs-i

**Fig. 3:**
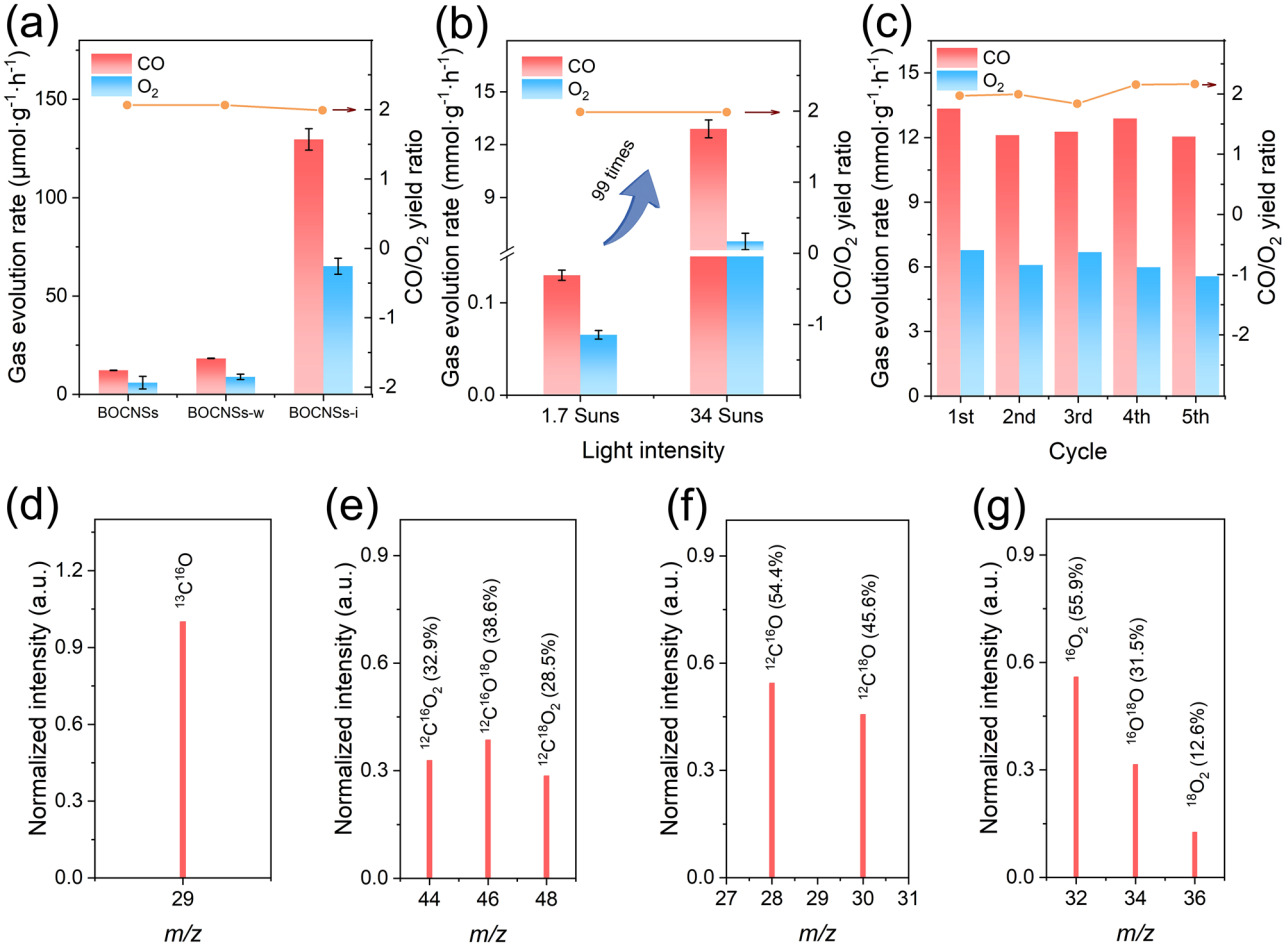
**a** Gas evolving rates of BOCNSs, BOCNSs-w, and BOCNSs-i, **b** gas evolving rates of BOCNSs-i under irradiation with different light intensities, and **c** cycling tests of photocatalysis performances for 5 times. **d** Mass spectrum of CO gas evolved during photocatalytic ^13^CO_2_ (99%) overall splitting over BOCNSs-i with H_2_O vapor fed. Mass spectra of **e** CO_2_, **f** CO, and **g** O_2_ after photocatalytic CO_2_ overall splitting over BOCNSs-i with H_2_^18^O vapor fed

**Fig. 4:**
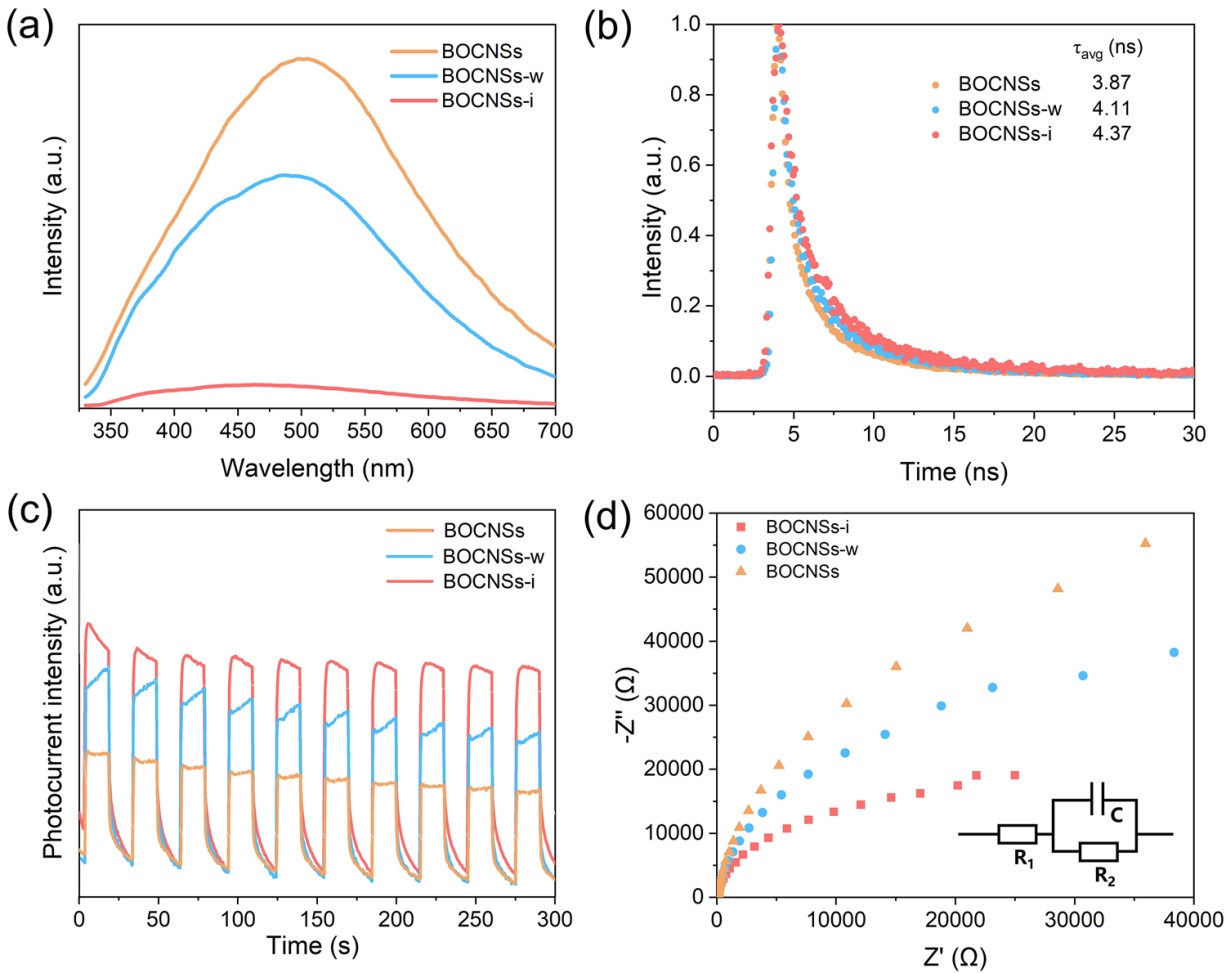
**a** PL spectra, **b** time-resolved transient PL decay spectra, **c** transient photocurrent curves, and **d** electrochemical impedance spectra of BOCNSs, BOCNSs-w, and BOCNSs-i, inset: the equivalent circuit, R_1_, R_2_, and C represent bulk charge transport resistance, interfacial charge transfer resistance and capacitance, respectively

**Fig. 5:**
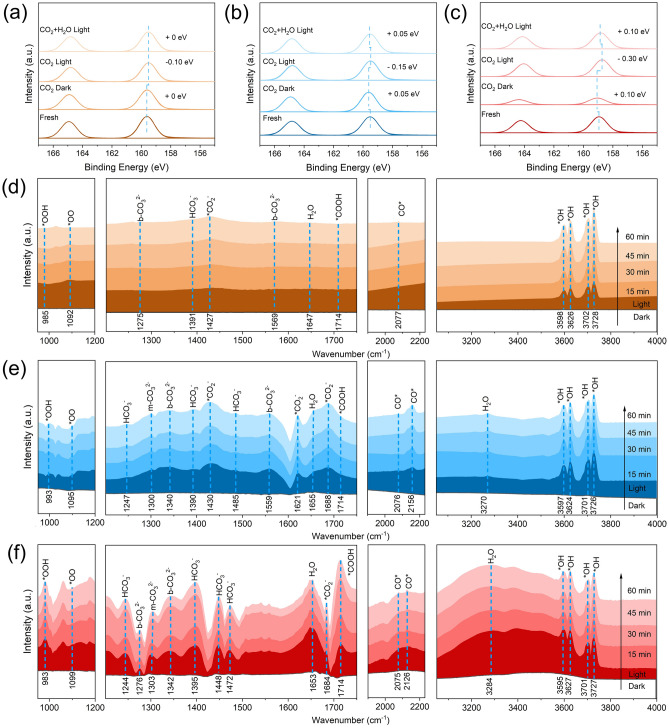
In situ XPS Bi 4*f* spectra of **a** BOCNSs, **b** BOCNSs-w, and **c** BOCNSs-i for photocatalytic CO_2_ overall splitting with or without H_2_O vapor fed. In situ DRIFTS spectra of **d** BOCNSs, **e** BOCNSs-w, and **f** BOCNSs-i for photocatalytic CO_2_ overall splitting with H_2_O vapor fed

BiOCl with layered or nanosheet structure (BOCNSs) obtained by hydrothermal method was exfoliated into ultrathin nanosheets (BOCNSs-w) and even atomic layers (BOCNSs-i) via ultrasonication treatment in water and isopropanol (Fig. 1a), respectively. Given the unique layered structure, all the obtained BiOCl samples exhibit a typical morphology of nanosheets with tetragonal phase, as identified by the (110) plane with lattice spacing of 2.75 Å (Fig. 1b–d) [17, 18]. With nanosheet structure and elemental distribution well maintained (Fig. S1–3), one would observe that the thickness is greatly reduced for both BOCNSs-w and BOCNSs-i as compared to BOCNSs (Fig. S4a-c). Detailed investigation into the nanosheet structure (Fig. 1e) reveals that BOCNSs-i is mainly exposed with (001) facets [19–21], at which Bi atoms are periodically arranged with the orthogonal (110) facet lattice fringe (Fig. 1f). Aberration-corrected high-angle annular dark-field scanning transmission electron microscopy (HAADF-STEM) image recorded along the [110] direction indicates that the Bi_2_O_2_ monolayers (Fig. 1g), interlacedly stacked with van der Waals gaps [19], are terminated with Bi atoms acting as the possible active sites for photocatalysis. The exfoliated nanosheet structures were then confirmed by AFM, with thickness significantly reduced from 8 to 9 nm for BOCNSs (Fig. 1h) to 4–5 nm for BOCNSs-w (Fig. 1i) and further to 2–3 nm for BOCNSs-i (Fig. 1j). This observation implies that isopropanol molecules are more likely than water molecules to be intercalated into the layered structure and exfoliate BOCNSs into atomic layers with increased exposure of (001) facets. In this case, Kelvin probe force microscopy (KPFM) measurements record that the surface potentials are gradually increased from 8.89 mV for BOCNSs to 21.74 mV for BOCNSs-w and further to 34.66 mV for BOCNSs-i (Fig. 1h–j*, insets*), depending on the decreasing nanosheet thickness. Along with the gradual increase in Zeta potentials (Fig. S5), the built-in electric field intensity is determined to be increased in the order of BOCNSs < BOCNSs-w < BOCNSs-i (Fig. S6) [22, 23], which would benefit charge carrier separation and then improve photocatalytic activity. Given the reduced nanosheet thickness, the specific surface areas of BiOCl are much increased after exfoliation (Fig. S7), contributing to the enhanced CO_2_ physical adsorption capacity (Fig. S8) and potentially the increased surface-active sites for BOCNSs-w and BOCNSs-i relative to BOCNSs.

The exfoliation triggered crystal structure evolution was then investigated by XRD patterns and Raman spectra. With all the BiOCl samples indexed to the tetragonal phase (PDF#06-0249) (Fig. 2a), the (001), (002), and (003) peaks are intensified significantly after exfoliation, indicating the increased exposure of (001) facets depending on the reduced thickness for the exfoliated BiOCl nanosheets, especially BOCNSs-i with only several atomic layers in thickness. Meanwhile, other XRD peaks are weakened or even vanished, signifying the dramatically decreased exposure of other facets along with the dominated exposure of (001) facets after exfoliation. In Raman spectra, three Raman bands are recorded at 60, 144, and 201 cm^−1^ for all the BiOCl samples, corresponding to the external A_1g_, A_1g_, and E_g_ stretching modes of Bi-Cl bonds (Fig. S9), respectively. With the A_1g_ and E_g_ Raman signals weakened for BOCNSs-w and BOCNSs-i as compared to BOCNSs, the external A_1g_ stretching signal is shifted to lower wavenumber and gets stronger for BOCNSs-i, revealing the increased amount of extended Bi–Cl bonds in atomic layers [15]. Furthermore, a new Raman band could be observed at 72 cm^−1^ for BOCNSs-i, assigned to the first-order vibration E_g_ mode of Bi metal, which well evidences the creation of V_O_ at the atomic layers during ultrasonication exfoliation in isopropanol [19, 24]. With the bandgaps of BOCNSs, BOCNSs-w, and BOCNSs-i determined to be 3.34, 3.26, and 3.21 eV (Fig. S10), respectively, the ultrasonication treatment would not significantly change the band structures and density of states of BiOCl (Figs. S11–S13). Interestingly, both BOCNSs-w and BOCNSs-i are featured with apparent Urbach tails in visible light region, which again confirms the introduction of defects in these exfoliated BiOCl (i.e., BOCNSs-w and BOCNSs-i) [25]. More likely, V_O_ is created at these two exfoliated BiOCl samples, especially, BOCNSs-i, given the instability of oxygen atoms exposed on the surface of BiOCl atomic layers [9], as further supported by ESR spectra. A single Lorentzian line centered at *g* = 2.005, the signal of V_O_ [26], is much increased for BOCNSs-w and BOCNSs-i (Fig. 2b), suggesting the introduction of V_O_ into BiOCl during exfoliation. The highest ESR signal noted for BOCNSs-i reveals the abundant V_O_ introduced in the exfoliated atomic layers. To deepen the understanding of electronic and coordination structure evolution triggered by liquid-phase exfoliation for BiOCl, X-ray absorption spectroscopy (XAS) measurements were conducted. Bi L_3_-edge XANES spectra present the very similar adsorption profiles for all the BiOCl samples, close to Bi_2_O_3_ [27], indicating that the chemical valence state of Bi in BiOCl samples should be + 3 (Fig. 2c). In details, the absorption intensity is gradually increased for BOCNSs exfoliated in water and isopropanol (Fig. 2c*,* inset), suggesting the increased electron densities at Bi atoms in the order of BOCNSs < BOCNSs-w < BOCNSs-i. The atomic coordination conditions were further examined by Bi R-space EXAFS spectra. A Bi-O peak located at 1.71 Å could be identified for all the BiOCl samples (Fig. 2d), with intensities gradually decreased from BOCNSs to BOCNSs-w and further to BOCNSs-i (Fig. 2d*,* inset), suggesting the unsaturated Bi coordination caused by the V_O_ introduced into the exfoliated ultrathin and atomic layers [12, 19]. All these observations and analysis indicate that Vo is introduced into BiOCl ultrasonically exfoliated in water and isopropanol, especially for BOCNSs-i, with electrons enriched Bi sites exposed on the exfoliated atomic layers.

### Photocatalytic Performances for CO_2_ Overall Splitting

Photocatalytic activity for CO_2_ overall splitting over these obtained BiOCl samples was evaluated on a homemade gas–solid reactor fed with CO_2_ gas and H_2_O vapor under the illumination of 300 W Xe lamp (1.7 suns) (Fig. S14). For all the BiOCl samples, the main products are CO and O_2_ with molar ratio determined to be 2:1, suggesting CO_2_ overall splitting: CO_2_ → CO + 1/2O_2_. BOCNSs could produce CO at a low rate of only 12.3 µmol g^−1^ h^−1^. In comparison, BOCNSs-w exhibits a slightly increased photocatalytic activity for CO_2_ overall splitting, with CO evolution rate reaching 18.4 µmol g^−1^ h^−1^. Excitingly, the photocatalytic activity is remarkably enhanced for BOCNSs-i, with CO evolution rate increased up to 134.8 µmol g^−1^ h^−1^, ~ 11 times that of BOCNSs (Fig. 3a). Such a high activity has surpassed those of the state-of-the-art Bi_l_O_m_X_n_ (X: Cl, Br, I) based photocatalysts (Table S1). It is noteworthy that under concentrated light irradiation (34 suns) with light intensity increased by 20 times, the CO evolution rate could be increased by ~ 99 times for BOCNSs-i, reaching 13.3 mmol g^−1^ h^−1^ as high (Fig. 3b). This dramatic increase in photocatalytic activity under concentrated light irradiation should be attributed to the enhanced photoexcitation by excluding the photothermal effect (Fig. S15). Note the well-maintained gas production rates and product ratios during 5-cycle photocatalytic reactions (Fig. 3c), BOCNSs-i holds excellent stability for photocatalytic CO_2_ overall splitting even under highly concentrated light irradiation, as evidenced by the almost unchanged crystal structures (Fig. S16), implying its great promise in solar-driven CO_2_ conversion into value-added fuels and chemicals.

To ravel out the fundamental reactions of photocatalysis for CO_2_ overall splitting, control and isotope labeling experiments were conducted over BOCNSs-i under various reaction conditions. No product could be detected with CO_2_ gas replaced by N_2_, in dark or without photocatalyst (Fig. S17), inferring that CO should originate from CO_2_ via photocatalysis process driven by BOCNSs-i. ^13^CO_2_ isotope experiment further confirms that CO is only produced from CO_2_ (Figs. 3d and S18). Interestingly, without H_2_O vapor fed into the reactor, BOCNSs-i exhibits a significantly reduced CO evolution rate. This comparative result indicates that H_2_O molecules could accelerate the CO_2_ overall splitting process, with detailed reactions revealed by C^16^O_2_ and H_2_^18^O isotope labeling experiments. Surprisingly, both ^16^O and ^18^O labeled CO_2_ could be recorded in the mass spectra of CO_2_ as the resource for CO production (Fig. 3e), i.e., C^16^O_2_ (m/z = 44), C^16^O^18^O (m/z = 46) and C^18^O_2_ (m/z = 48), during photocatalytic reaction. It could be thus reasonably supposed that the ^18^O atom in H_2_^18^O molecules would exchange with the ^16^O atom in C^16^O_2_ molecules, by following the reactions: C^16^O_2_ + H_2_^18^O → C^16^O^18^O + H_2_^16^O, and C^16^O^18^O + H_2_^18^O → C^18^O_2_ + H_2_^16^O. In consequence, ^16^O and/or ^18^O labeled CO (C^16^O at m/z = 28, C^18^O at m/z = 30) (Fig. 3f) and O_2_ (^16^O_2_ at m/z = 32, ^16^O^18^O at m/z = 34, ^18^O_2_ at m/z = 36) (Fig. 3g) are produced via CO_2_ overall splitting: C^16^O_2_ → C^16^O + 1/2^16^O_2_, C^16^O^18^O → C^18^O + 1/2^16^O_2_, C^16^O^18^O → C^16^O + 1/2^16^O^18^O, and C^18^O_2_ → C^18^O + 1/2^18^O_2_. These analytic results rationalize the important role of H_2_O vapor fed during photocatalytic reaction, with O atoms exchanged between H_2_O and CO_2_, accelerating CO_2_ overall splitting reactions by regulating the intermediate behaviors and the reaction pathways as experimentally and theoretically proved in the following discussions.

### Charge Carrier Transfer Properties

To rationally unravel the reasons for the much improved photocatalytic activity for CO_2_ overall splitting over the exfoliated BiOCl nanosheets, optical and electrochemical measurements were conducted to explore the photogenerated charge transfer properties. Steady-state PL spectra display that the distinct PL emission observed at 500 nm, originated from the conduction-to-valence band transition, is gradually quenched for BOCNSs-w and further for BOCNSs-i as compared to BOCNSs (Fig. 4a). Moreover, as revealed in TRPL spectra (Fig. 4b), the average lifetime of photogenerated carriers is prolonged from 3.87 ns for BOCNSs to 4.11 ns for BOCNSs-w and further to 4.37 ns for BOCNSs-i. Both the attenuated PL emission quenching and the prolonged average carrier lifetime indicate that the photogenerated charge transfer is effectively promoted in the order of BOCNSs < BOCNSs-w < BOCNSs-i, attributed to the shortened charge diffusion distance and the strengthened built-in electric fields (Fig. S6) in the exfoliated BiOCl nanosheets with reduced thickness. This improved charge transfer ability could be also confirmed by the increased transient photocurrent density (Fig. 4c) and the decreased charge carrier transfer resistance (Table S2) determined from electrochemical impedance spectra (Fig. 4d).

### Photocatalytic Mechanisms for CO_2_ Overall Splitting

Given the much improved photocatalytic activity obtained over exfoliated BiOCl, especially BOCNSs-w, in situ spectral investigations were conducted to reveal the mechanistic fundamentals of photocatalytic CO_2_ overall splitting. In situ XPS was performed to identify the active sites and monitor the electronic interaction between active sites and reactants. For fresh BOCNSs, the Bi 4*f* XPS spectrum displays two peaks of Bi 4*f*_7/2_ and Bi 4*f*_5/2_ at 159.60 and 164.90 eV (Fig. 5a), respectively, assigned to Bi^3+^ in BiOCl [28]. A negligible shift in the Bi 4*f* peaks could be observed with BOCNSs exposed in CO_2_ for saturated adsorption in dark, indicating the disable activation of CO_2_ molecules at Bi sites. Under irradiation, the Bi 4*f* peaks are shifted to lower binding energy by 0.1 eV, implying the accumulation of electrons at Bi sites upon photoexcitation. However, the fed H_2_O vapor does not cause further shift in the binding energy of Bi 4*f* orbitals, implying the ignorable interaction between Bi sites and H_2_O molecules. Such electronic inertness of CO_2_ and H_2_O at Bi sites in BOCNSs should be responsible for its poor photocatalytic activity for CO_2_ reduction. In comparison, the Bi 4*f* peaks are positively shifted by 0.05 eV for BOCNSs-w exposed in CO_2_ for saturated adsorption in dark, Fig. 5b, due to the chemical adsorption of CO_2_ at surface with electrons transfer from Bi sites to CO_2_ molecules. A 0.15 eV negative shift in the Bi 4*f* peaks could be then noted under irradiation, and subsequently, a 0.05 eV positive shift happens with H_2_O vapor fed into CO_2_ atmosphere. For BOCNSs-i, the Bi 4*f*_7/2_ and Bi 4*f*_5/2_ peaks are located at 158.90 and 164.20 eV, respectively, more negative than BOCNSs and BOCNSs-w, which indicates the higher electron density at Bi sites in BOCNSs-i, agreeing well with the analysis in Bi L_3_-edge XANES. Further note the more distinct shifts in the Bi 4*f* peaks happened to BOCNSs-i exposed in CO_2_ for saturated adsorption in dark (+ 0.10 eV), under irradiation (− 0.30 eV) and then fed with H_2_O vapor (+ 0.10 eV) (Fig. 5c), evidencing the more efficient adsorption and activation of CO_2_ molecules at electrons enriched Bi sites. These observable XPS peak shifts could thus claim that for these exfoliated BiOCl with reduced thickness and V_O_ introduced at surface, especially BOCNSs-i, the exposed Bi sites would be offered with plentiful electrons upon photoexcitation and then transfer these electrons to activate the adsorbed CO_2_ molecules under the assistance of H_2_O molecules. This activation effect induced by H_2_O molecules may alter the reaction intermediate behaviors and then accelerate the subsequent CO_2_ overall splitting reaction by reducing the energy barrier of RDS.

In situ DRIFTS was then conducted to monitor the reaction intermediates and reveal the reaction pathways for photocatalytic CO_2_ overall splitting over BiOCl samples. It is clear that, for the three BiOCl samples during photocatalytic CO_2_ overall splitting with H_2_O vapor fed, a series of infrared bands, such as *CO_2_^−^ (1684 ~ 1688, 1621, 1427 ~ 1430 cm^−1^), m-CO_3_^2−^ (1300 ~ 1303 cm^–1^), b-CO_3_^2−^ (1559 ~ 1569, 1340 ~ 1342, 1275 ~ 1276 cm^−1^), HCO_3_^−^ (1472 ~ 1485, 1448, 1390 ~ 1395, 1244 ~ 1247 cm^−1^), H_2_O, (1647 ~ 1655, 3100 ~ 3500 cm^−1^), *OH (3595 ~ 3728 cm^−1^), and *COOH (1714 cm^−1^), could be observed, with intensities gradually increased depending on the prolonging irradiation time (Fig. 5d–f) [29–36]. The signals of *CO_2_^−^ species for BOCNSs-w (1688, 1621, and 1430 cm^−1^) are more distinct than BOCNSs (1427 cm^−1^), indicating the improved CO_2_ activation ability over BOCNSs-w. However, the *CO_2_^–^ peak at 1684 cm^−1^ is much weakened for BOCNSs-i, probably due to the fast consumption of CO_2_ in the subsequent CO_2_ reduction processes [29–31]. The broad bands located at 3100–3500 and 1647–1655 cm^−1^, assigned to the adsorbed H_2_O molecules [31, 32], are stronger for BOCNSs-i than BOCNSs-w, while almost absent over BOCNSs. This observation indicates that the exfoliated BiOCl samples, especially BOCNSs-i, could provide abundant sites for adsorption of H_2_O molecules, due to the activated electronic interaction between H_2_O molecules and electrons enriched Bi sites. These surface-adsorbed H_2_O molecules would then split and produce *OH species (3595–3728 cm^–1^) [33, 34], which further react with *CO_2_^−^ to form hydrated CO_2_ species (*OH + *CO_2_^−^ → HCO_3_^−^) [30]. The peak associated with HCO_3_^–^ species could be observed at 1390–1395 cm^−1^ for BOCNSs, BOCNSs-w, and BOCNSs-i [35], which is believed to play the role of key intermediate for CO_2_ overall splitting. In addition, careful comparison tells that the HCO_3_^–^ signals at ca. 1395 cm^−1^ for BOCNSs-i are stronger than BOCNSs and even BOCNSs-w, with additional HCO_3_^−^ signals at 1472, 1448, and 1244 cm^−1^ much weaker for BOCNSs-w and even invisible for BOCNSs [29, 37], which indicates the favorable generation of HCO_3_^−^ species at BOCNSs-i, accounting for the rapid consumption of *CO_2_^–^ species. The band at 1714 cm^−1^ corresponding to *COOH species could be observed more distinctly for BOCNSs-i than BOCNSs-w and BOCNSs [29, 36], suggesting the superior generation of *COOH species along with O* intermediate from the HCO_3_^−^ species previously formed on BOCNSs-i (HCO_3_^−^ → *COOH + *O) [38]. Additionally, the signals assigned to the surface-adsorbed *CO species could be observed at 2070–2160 cm^−1^ [30, 39], much stronger for BOCNSs-i than BOCNSs and even BOCNSs-w, which indicates the superior formation of *CO intermediates (*COOH → *OH + *CO) on BOCNSs-i for efficient CO generation. The peaks at 983–993 cm^–1^, which are related to the *OOH species, the key intermediate for O_2_ evolution and generated via the coupling of *OH and *O (*OH + *O → *OOH), are more distinct for BOCNSs-i than BOCNSs-w, while hardly observed for BOCNSs. Further careful comparison on the infrared signals of *OO species located at 1092–1099 cm^−1^ reveals that the accumulation of *OO species (*OOH + *OH → *OO + H_2_O) are more significant over BOCNSs-i than BOCNSs-w and BOCNSs [40], which evidences the favorable O_2_ evolution over BOCNSs-i via CO_2_ overall splitting. These in situ spectral analytic results, together with in situ Raman investigations (Fig. S19), indicate that the electrons enriched Bi sites in BOCNSs-i would activate the adsorbed CO_2_ molecules under the assistance of H_2_O vapor fed to accelerate CO_2_ overall splitting reactions.

**Fig. 6:**
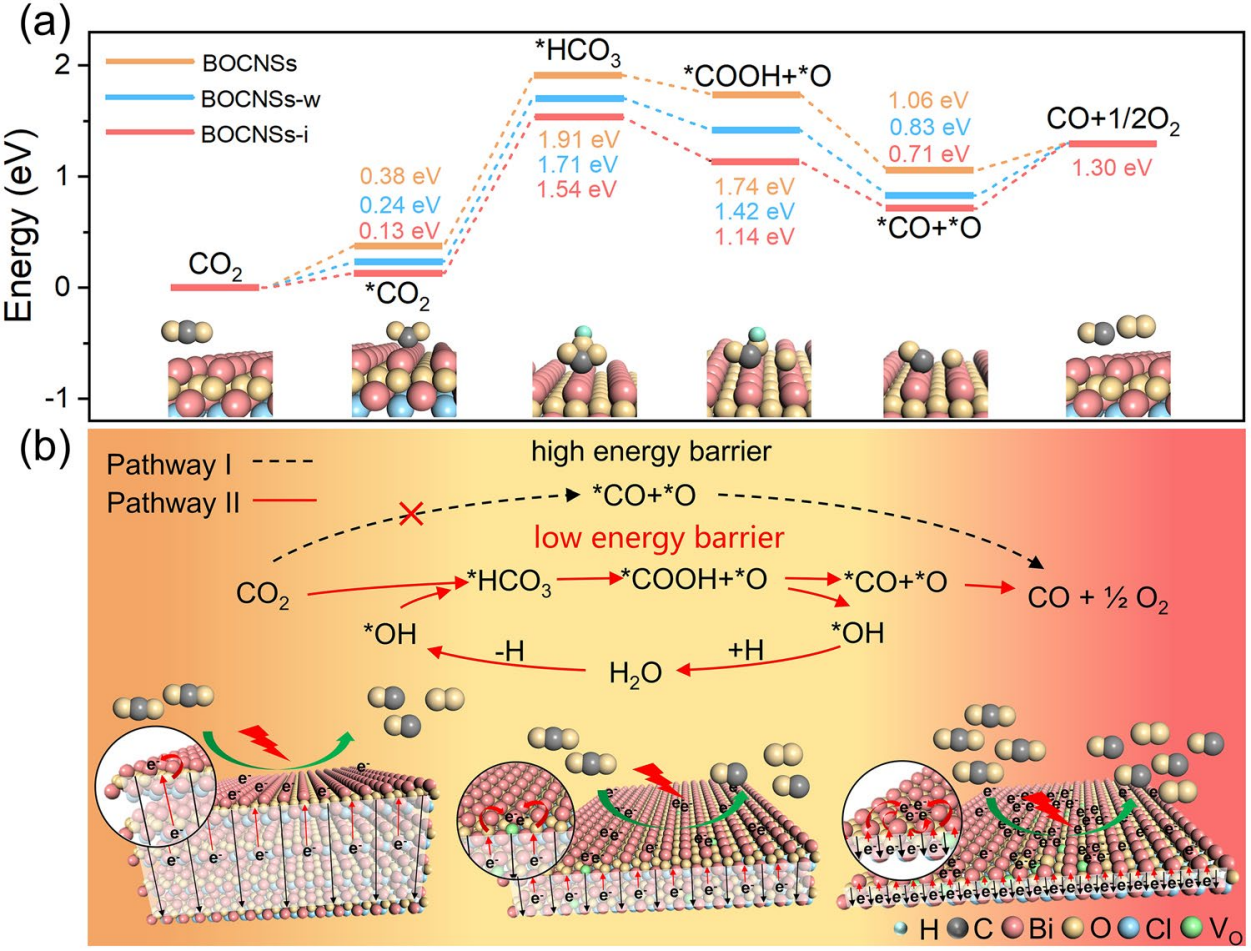
**a** Energetic reaction pathways and **b** proposed mechanism for photocatalytic CO_2_ overall splitting over BOCNSs, BOCNSs-w, and BOCNSs-i

To reveal the important role of H_2_O vapor fed in the process of CO_2_ overall splitting, with crystal structures optimized for BOCNSs-i (Fig. S11c), DFT calculations were conducted to investigate energetic pathways of photocatalytic CO_2_ overall splitting over BOCNSs-i with or without H_2_O fed (Fig. S20). For photocatalytic CO_2_ overall splitting without H_2_O vapor fed, with reaction pathway reasonably determined to be *CO_2_^−^ → *CO + *O → CO + O_2_ (Pathway I) (Fig. S20a) [41], the splitting of surface-adsorbed *CO_2_^−^ into *CO and *O, i.e., *CO_2_^−^ → *CO + *O, should act as the RDS, requiring a large energy barrier of 2.51 eV, which well explains the poor CO evolution rate. In comparison, in the presence of H_2_O vapor, the reaction pathway for photocatalytic CO_2_ overall splitting could be theoretically proposed as *CO_2_^−^ → HCO_3_^−^ → *COOH + *O → *CO + *O → CO + O_2_ (Pathway II) (Fig. S20b), which has been well evidenced by the in situ DRIFTS investigations with all these intermediates clearly identified. Specifically, H_2_O molecules react with CO_2_ to form HCO_3_^−^, which would be converted to *COOH and *O by breaking the C-O bonds. Then, the formed *COOH intermediates would split into *OH and *CO for CO generation. Note the generation of HCO_3_^–^ intermediates (i.e., *CO_2_^−^ → HCO_3_^−^) regarded as the RDS for Pathway II, which requires a free energy difference of 1.41 eV, much smaller than that of the RDS for Pathway I (2.51 eV). The photocatalytic CO_2_ overall splitting is much more favorable with H_2_O vapor fed than in the absence of H_2_O vapor. Next, to investigate the mechanisms behind the superior performance for BOCNSs-i, the Gibbs free energy of elementary steps for photocatalytic CO_2_ overall splitting over BOCNSs (Fig. S11a), BOCNSs-w (Fig. S11b), and BOCNSs-i (Fig. S11c) with H_2_O fed was theoretically explored by DFT calculations. It is noteworthy that the Gibbs free energy for the generation of various key intermediates (*CO_2_^−^, HCO_3_^−^, *COOH, *CO, and *O) is much lower for BOCNSs-i than BOCNSs and even BOCNSs-w (Fig. 6a). Especially, for the RDS of *CO_2_^−^ → HCO_3_^−^, the energy barrier required by BOCNSs-i (1.41 eV) is significantly lower than BOCNSs (1.53 eV) and BOCNSs-w (1.47 eV). These comparative results demonstrate that BOCNSs-i is more conducive than BOCNSs and BOCNSs-w to converting surface-adsorbed *CO_2_^−^ to HCO_3_^−^, as well supported by the in situ DRIFTS investigations, which contributes to its superior performance for photocatalytic CO_2_ overall splitting.

Based on the aforementioned theoretical calculations and experimental characterizations, the mechanism of photocatalytic CO_2_ overall splitting with H_2_O vapor fed over BOCNSs-i could be reasonably proposed (Fig. 6b). With thickness decreased, the built-in electric field intensity is significantly increased, and the diffusion distance of photogenerated carriers is shortened for BOCNSs-i as compared to BOCNSs and BOCNSs-w, which benefits the charge carrier transfer for efficient photocatalysis. Moreover, with V_O_ introduced into the atomic layers of BOCNSs-i, the electrons enriched Bi active sites would transfer electrons to activate CO_2_ and H_2_O molecules, reducing the energy barrier of RDS. We should note that the reaction pathway for photocatalytic CO_2_ overall splitting would be switched from *CO_2_^–^ → *CO + *O → CO + O_2_ (Pathway I) in the absence of H_2_O vapor to *CO_2_^–^ → HCO_3_^–^ → *COOH + *O → *CO + *O → CO + O_2_ (Pathway II) under the assistance of H_2_O molecules exchanging oxygen atoms with CO_2_ gas, with the energy barrier of RDS greatly reduced for high-performance photocatalytic CO_2_ overall splitting.

## Conclusion

Starting with hydrothermally synthesized BiOCl with layered structure (BOCNSs), exfoliated BiOCl ultrathin nanosheets (BOCNSs-w) and even atomic layers (BOCNSs-i) were obtained via ultrasonication in water and isopropanol, respectively. With thickness greatly reduced and built-in electric field significantly increased for efficient charge carrier transfer and separation, BOCNSs-i exhibits a superior performance for photocatalytic CO_2_ overall splitting to produce CO and O_2_ at a stoichiometric ratio of 2:1, with CO evolution rate reaching 134.8 µmol g^−1^ h^−1^ under simulated solar light (1.7 suns), which is 11.0 and 7.3 times that of BOCNSs and BOCNSs-w, respectively. Moreover, the CO evolution rate is further increased up to 13.3 mmol g^−1^ h^−1^ under concentrated solar irradiation (34 suns), 99 times that under simulated solar light (1.7 suns), indicating that BOCNSs-i could serve as one of the promising candidates for photocatalytically converting CO_2_ to value-added chemicals. In situ spectral investigations and theoretical calculations reveal that electrons enriched Bi active sites exposed at the atomic layers of BOCNSs-i would transfer electrons to activate CO_2_ and H_2_O molecules and lower the energy barrier of RDS for photocatalytic CO_2_ overall splitting. Moreover, isotope label experiments verify the exchange process of oxygen atoms between H_2_O and CO_2_ molecules during CO_2_ overall splitting reaction with H_2_O vapor fed, which could reduce the energy barrier of RDS by altering the reaction pathways, resulting in the encouraging photocatalytic activity. This research provides an inspiration to rational design of high-performance photocatalyst for CO_2_ overall splitting, with electronic and atomic fundamentals well explored to deepen the mechanistic understandings of solar-driven CO_2_ photocatalysis.

## Supplementary Information

Below is the link to the electronic supplementary material.

Supplementary file1 (DOCX 0 KB)
