# Supplementary material for: BiOCl Atomic Layers with Electrons Enriched Active Sites Exposed for Efficient Photocatalytic CO2 Overall Splitting
Source: Nanomicro Lett. 2025 Apr 18;17:223. doi: 10.1007/s40820-025-01723-2 (PMC12008097; doi:10.1007/s40820-025-01723-2)
Supplement: Supplementary file 1 — Supplementary file1 (DOCX 0 KB) [file 40820_2025_1723_MOESM1_ESM.docx]

Supporting Information for

**BiOCl Atomic Layers with Electrons Enriched Active Sites Exposed for Efficient Photocatalytic CO_2_ Overall Splitting**

Ting Peng^1+^, Yiqing Wang^1+^, Chung-Li Dong^2^, Ta Thi Thuy Nga^2^, Binglan Wu^1^, Yiduo Wang^1^, Qingqing Guan^1^, Wenjie Zhang^1^, and Shaohua Shen^1^*

^1^International Research Center for Renewable Energy, State Key Laboratory of Multiphase Flow in Power Engineering, Xi’an Jiaotong University, Xi’an 710049, P. R. China

^2^Department of Physics, Tamkang University, New Taipei City 25137, Taiwan, P. R. China

^+^ Ting Peng and Yiqing Wang contributed equally to this work.

*Corresponding author. E-mail: [shshen_xjtu@mail.xjtu.edu.cn](mailto:shshen_xjtu@mail.xjtu.edu.cn) (Shaohua Shen)

**Supplementary Figures**

**
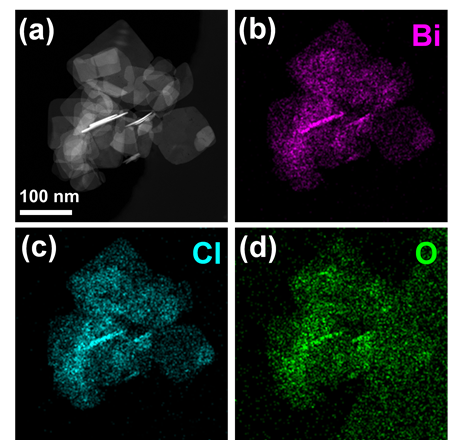
**

**Fig. S1** (**a**) HAADF-STEM image, and elemental mappings of (**b**) Bi, (**c**) Cl, and (**d**) O of BOCNSs


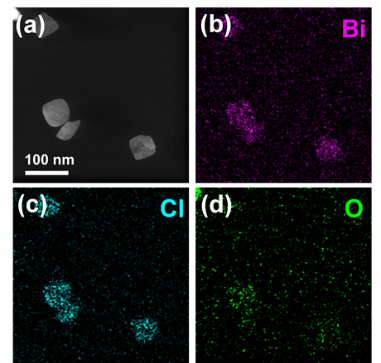


**Fig. S2** (**a**) HAADF-STEM image, and elemental mappings of (**b**) Bi, (**c**) Cl, and (**d**) O of BOCNSs-w


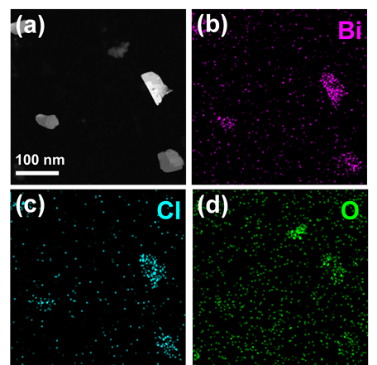


**Fig. S3** (**a**) HAADF-STEM image, and elemental mappings of (**b**) Bi, (**c**) Cl, and (**d**) O of BOCNSs-i


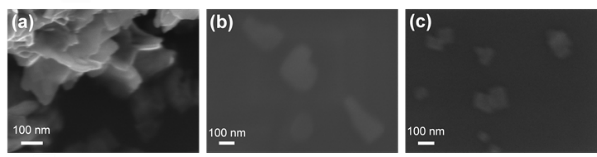


**Fig. S4** SEM images of (**a**) BOCNSs, (**b**) BOCNSs-w, and (**c**) BOCNSs-i

**Fig. S5** Zeta potentials of BOCNSs, BOCNSs-w and BOCNSs-i

**Fig. S6** Normalized built-in electric field intensities of BOCNSs, BOCNSs-w and BOCNSs-i

The normalized built-in electric field intensities (F) for the three samples were calculated from surface potentials (V_s_) (Fig. 1h-j*, insets*) and zeta potentials (ζ) (Fig. S5) by a function of surface potential and Zeta potential [S1-S3].

$\text{F}\text{=}\left( \text{-}\text{2}\left( \frac{\text{8kTn}}{\text{ε}\text{ε}_{\text{0}}} \right)^{\text{1/2}}\text{V}_{\text{s}}\sin\text{h}\left( \frac{\text{Z}\text{e}_{\text{0}}\text{ζ}\left( \text{1+ }\frac{\text{D}}{\text{a}_{\text{1}}} \right)\text{e}^{\text{κ}\text{D}}}{\text{2kT}} \right) \right)^{\text{1}\text{/2}}$ (1)

k: Boltzmann constant, J∙K^−1^

T: Absolute temperature, K

n: Number of electrolytes per unit volume, m^−3^

e_0_: Electron charge, C

sinh: Hyperbolic sine function

Z: Electrolyte valence

a_1_: Particle Stokes radius, m

𝜅^−1^: Debye length, nm

D: Distance from sliding layer to particle surface, Å

𝜀: Relative medium constant

𝜀_0_: vacuum dielectric constant, F∙m^−1^

As shown in Fig. S6, the normalized built-in electric field intensity calculated for BOCNSs-w and BOCNSs-i is 2.6 times and 6.3 times that of BOCNSs, respectively.

**Fig. S7** Specific surface areas of BOCNSs, BOCNSs-w, and BOCNSs-i

**Fig. S8** CO_2_ physical adsorption isotherms of BOCNSs, BOCNSs-w, and BOCNSs-i

**Fig. S9** Raman spectra of BOCNSs, BOCNSs-w, and BOCNSs-i

**Fig. S10** (**a**) UV–vis diffuse reflectance spectra (UV-vis DRS) and (**b**) the first derivative of UV-vis DRS of BOCNSs, BOCNSs-w, and BOCNSs-i

The lowest value of *x*-axis (X) in the first derivative of UV-vis DRS (Fig. S10a) is determined to be 352.12, 346.10 and 347.19 for BOCNSs, BOCNSs-w, and BOCNSs-i (Fig. S10b), respectively. By making a tangent at the point of UV-vis DRS spectra corresponding to X value (Fig. S10a), and the intercept point at x-axis is 371.68 nm, 380.71 nm and 386.05 nm, respectively, which is the absorption edge (λ_g_) of BOCNSs, BOCNSs-w, and BOCNSs-i. The band gap (E_g_) could be then calculated by Eq. (S2) to be 3.34 eV, 3.26 eV, and 3.21 eV for BOCNSs, BOCNSs-w, and BOCNSs-i, respectively.

E_g_ =1240 / λ_g_ (S2)

**Fig. S11** Optimized structural models of (**a**) BOCNSs, (**b**) BOCNSs-w and (**c**) BOCNSs-i

Structural characterizations confirm the tetragonal structure of BiOCl with exposed (001) facet for all the three samples. Note the reduced thickness and increased oxygen vacancy density for BOCNSs-w and BOCNSs-i as compared to BOCNSs. The structure models of four, two and one BiOCl layers exposed with (001) surface and terminated with Bi atoms were built for BOCNSs (Fig. S11a), BOCNSs-w (Fig. S11b) and BOCNSs-i (Fig. S11c) for theoretical calculations, respectively. Moreover, 36 and 72 oxygen vacancies were introduced into the structure models of BOCNSs-w and BOCNSs-i, respectively.


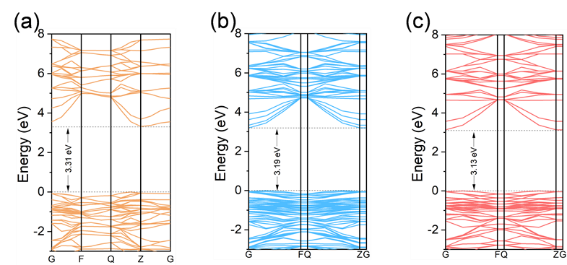


**Fig. S12** Calculated band structures of (**a**) BOCNSs, (**b**) BOCNSs-w, and (**c**) BOCNSs-i

As shown in Fig. S12, the calculated band structures are slightly narrowed from 3.31 eV for BOCNSs to 3.19 eV for BOCNSs-w and further to 3.13 eV for BOCNSs-i.

**Fig. S13** Density of states of (**a**) BOCNSs, (**b**) BOCNSs-w and (**c**) BOCNSs-i

As shown in Fig. S13, O 2p, Cl 3p and Bi 6p orbitals contribute to the conduction bands of BOCNSs, BOCNSs-w and BOCNSs-i, and their valence bands are mainly composed of Bi 6p orbitals. No insignificant change in the density of states could be observed for BiOCl after exfoliation.


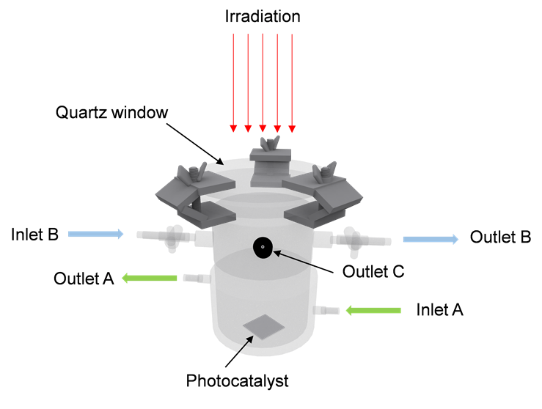


**Fig. S14** Schematic diagram of homemade gas-solid photocatalytic reactor

The volume of photocatalytic reactor (Fig. S14) is 130 mL. Cooling water was flowed into jacket at inlet A and out at outlet A. Air was evacuated by vacuum pump connected with outlet B. CO_2_ gas was conducted into reactor at inlet B. Light was irradiated onto the photocatalyst through the quartz window. Outlet C is a port for sampling.

**Fig. S15** Thermal images of (**a**) clean FTO glass and (**b**) FTO glass covered with BOCNSs-i

As shown in Fig. S15, the thermal images recorded for clean FTO and BOCNSs-i covered FTO display very similar temperatures under concentrated light irradiation (34 suns), which further implies that photothermal effect could be hardly generated by BiOCl. Thus, the dramatic increase in CO evolution rate over BOCNSs-i under the concentrated light irradiation (34 suns) should be attributed to the enhanced photoexcitation.

**Fig. S16** XRD patterns of BOCNSs-i before and after photocatalytic reaction

One would note the very similar XRD patterns for BOCNSs-i before and after photoreaction under concentrated light irradiation (Fig. S16), which indicates the well maintained crystal structure for BOCNSs-i during photocatalytic reaction.

**Fig. S17** Gas evolution rates of BOCNSs-i under various reaction conditions


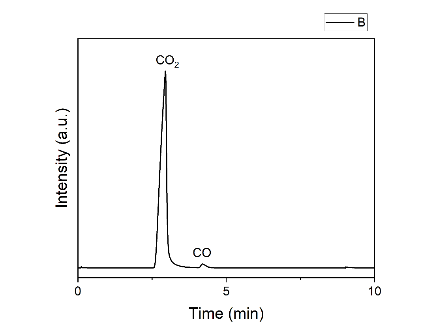


**Fig. S18** GC chromatogram recorded for photocatalytic ^13^CO_2_ (99%) overall splitting over BOCNSs-i with H_2_O vapor fed

The GC chromatogram exhibits two peaks at the retention time of 2.8 and 4.2 min (Fig. S18), related to CO_2_ and CO, respectively, which supports that the ^13^CO product is generated from ^13^CO_2_.

**Fig. S19** In-situ Raman spectra of (**a**) BOCNSs, (**b**) BOCNSs-w and (**c**) BOCNSs-i

In-situ Raman spectra were conducted to detect the intermediates. As shown in Fig. S19, the Raman band detected at ca. 1540 cm^–1^, assigned to the *CO_2_^–^ species, are more distinct for BOCNSs-i than BOCNSs (1538 cm^–1^) and BOCNSs-w (1540 cm^–1^) [S4, S5], indicating the favorable adsorption of CO_2_ molecules over BOCNSs-i. Meanwhile, the Raman bands at 1635~1640 cm^–1^ associated with the *COOH species [S6] are observed to be strengthened for BOCNSs-i than BOCNSs and BOCNSs-w, indicating the promoted *CO_2_^–^-to-*COOH conversion. Moreover, a careful comparison unveils that two small bands of *CO species at 2023 and 2107 cm^–1^ are detected on BOCNSs-i [S7], which are stronger than BOCNSs and BOCNSs-w, suggesting the superior formation of *CO intermediates on BOCNSs-i.


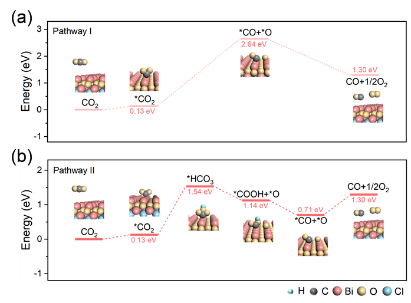


**Fig. S20** Energetic reaction pathways of photocatalytic CO_2_ overall splitting over BOCNSs-i (**a**) without and (**b**) with H_2_O fed

**Table S1** A comparison of Bi_l_O_m_X_n_ (X: Cl, Br, I) based photocatalysts for CO_2_ photoreduction. V_O_: oxygen vacancy, V_BiO_: bismuth-oxygen vacancy pairs, V_Cl_: chlorine vacancy, V_Bi_: bismuth vacancy.

| Catalyst | Main Products | Product yield  (µmol·g^-1^·h^-1^) | References |
| --- | --- | --- | --- |
| V_O_-PbBiO_2_Cl | CO | 16.02 | [S8] |
| Pb_0.6_Bi_1.4_O_2_Cl_1.4_ | CO, CH_3_OH, O_2_ | 4.48,6.63,12.17 | [S9] |
| BiOCl@Bi_2_O_3_ | CO | 75 | [S10] |
| V_O_-BiOIO_3_ | CO | 17.33 | [S11] |
| BiOIO_3_ | CO | 5.42 | [S12] |
| Co-Bi_3_O_4_Br | CO | 107.1 | [S13] |
| V_BiO_-Bi_24_O_31_Br_10_ | CO | 24.9 | [S14] |
| V_Cl_-Bi_4_O_5_Cl_2_ | CO | 14.62 | [S15] |
| V_O_-BiOBr | CO | 87.4 | [S16] |
| V_O_-Bi_12_O_17_Cl_2_ | CO | 48.6 | [S17] |
| V_Cl_-Bi_5_O_7_Cl | CO | 27.15 | [S18] |
| Bi_2_O_2_(OH)(NO_3_) | CO | 8.12 | [S19] |
| V_Bi_-BiOBr | CO | 20.1 | [S20] |
| V_Bi_-BiOCl | CO | 21.99 | [S21] |
| C_3_N_4_/BiOCl-V_O_ | CO, CH_4_ | 4.73, 0.77 | [S22] |
| BiOCl | CO, CH_4_ | 78.07, 3.03 | [S23] |
| BiOCl-B-V_O_ | CO | 83.64 | [S24] |
| Au–BiOCl-V_O_ | CO, CH_4_ | 3.46, 1.39 | [S25] |
| N-BiOCl | CO | 92.8 | [S26] |
| BOCNSs-i | CO, O_2_ | 134.80, 67.51 | This work |

**Table S2** EIS fitting parameters of the obtained BiOCl samples. R_1_, bulk charge transport resistance; R_2_, interfacial charge transfer resistance

| Sample | R_1_/Ω·cm^2^ | C/F·cm^-2^ | R_2_/Ω·cm^2^ |
| --- | --- | --- | --- |
| BOCNSs | 40.13 | 1.49×10^-5^ | 1.03×10^5^ |
| BOCNSs-w | 39.94 | 1.48×10^-5^ | 6.57×10^4^ |
| BOCNSs-i | 39.62 | 1.47×10^-5^ | 3.26×10^4^ |

**Supplementary References**

1. Y. Guo, Q. Zhou, J. Nan, W. Shi, F. Cui et al., Perylenetetracarboxylic acid nanosheets with internal electric fields and anisotropic charge migration for photocatalytic hydrogen evolution. Nat. Commun. **13**, 2067 (2022). <https://doi.org/10.1038/s41467-022-29826-z>
2. L. Zhai, X. She, L. Zhuang, Y. Li, R. Ding et al., Modulating built-in electric field via variable oxygen affinity for robust hydrogen evolution reaction in neutral media. Angew. Chem. Int. Ed. **134**, e202116057 (2022). <https://doi.org/10.1002/anie.202116057>
3. X. Zhao, M. Liu, Y. Wang, Y. Xiong, P. Yang et al., Designing a built-in electric field for efficient energy electrocatalysis. ACS Nano **16**(12), 19959–19979 (2022).. <https://doi.org/10.1021/acsnano.2c09888>
4. I. Chernyshova, P. Somasundaran, S. Ponnurangam, On the origin of the elusive first intermediate of CO_2_ electroreduction. Proc. Natl. Acad. Sci. USA **115**, E9261-E9270 (2018). <https://doi.org/10.1073/pnas.1802256115>
5. M. Wang, H. Chen, M. Wang, J. Wang, Y. Tuo et al., Tuning C1/C2 selectivity of CO_2_ electrochemical reduction over in-situ evolved CuO/SnO_2_ heterostructure. Angew. Chem. Int. Ed. **62**, e202306456 (2023). <https://doi.org/10.1002/anie.202306456>
6. H. Yang, Y. Hu, J. Chen, M. Balogun, P. Fang et al., Intermediates adsorption engineering of CO_2_ electroreduction reaction in highly selective heterostructure Cu-based electrocatalysts for CO production. Adv. Energy Mater. **9**, 1901396 (2019). <https://doi.org/10.1002/aenm.201901396>
7. H. An, L. Wu, L. Mandemaker, S. Yang, J. Ruiter et al., Sub-second time-resolved surface-enhanced raman spectroscopy reveals dynamic CO intermediates during electrochemical CO_2_ reduction on copper. Angew. Chem. Int. Ed. **60**, 16576 – 16584 (2021). <https://doi.org/10.1002/anie.202104114>
8. B. Wang, W. Zhang, G. Liu, H. Chen, Y. Weng et al., Excited electron‐rich Bi^(3–x)+^ sites: a quantum well‐like structure for highly promoted selective photocatalytic CO_2_ reduction performance. Adv. Funct. Mater. **32**(35), 2202885 (2022). <https://doi.org/10.1002/adfm.202202885>
9. X. Feng, R. Zheng, C. Gao, W. Wei, J. Peng et al., Unlocking bimetallic active sites via a desalination strategy for photocatalytic reduction of atmospheric carbon dioxide. Nat. Commun. **13**, 2146 (2022). <https://doi.org/10.1038/s41467-022-29671-0>
10. L. Wang, X. Zhao, D. Lv, C. Liu, W. Lai et al., Promoted photocharge separation in 2D lateral epitaxial heterostructure for visible‐light‐Driven CO_2_ photoreduction. Adv. Mater. **32**(48), 2004311 (2020). <https://doi.org/10.1002/adma.202004311>
11. F. Chen, Z. Ma, L. Ye, T. Ma, T. Zhang et al., Macroscopic spontaneous polarization and surface oxygen vacancies collaboratively boosting CO_2_ photoreduction on BiOIO_3_ single crystals. Adv. Mater. **32**(11), 1908350 (2020). <https://doi.org/10.1002/adma.201908350>
12. F. Chen, H. Huang, L. Ye, T. Zhang, Y. Zhang et al., Thickness‐dependent facet junction control of layered BiOIO_3_ single crystals for highly efficient CO_2_ photoreduction. Adv. Funct. Mater. **28**(46), 1804284 (2018). <https://doi.org/10.1002/adfm.201804284>
13. J. Di, C. Chen, S. Yang, S. Chen, M. Duan et al., Isolated single atom cobalt in Bi_3_O_4_Br atomic layers to trigger efficient CO_2_ photoreduction. Nat. Commun. **10**, 2840 (2019). <https://doi.org/10.1038/s41467-019-10392-w>
14. J. Di, C. Chen, C. Zhu, R. Long, H. Chen et al., Surface local polarization induced by bismuth‐oxygen vacancy pairs tuning non‐covalent interaction for CO_2_ photoreduction. Adv. Energy. Mater. **11**(41), 2102389 (2021). <https://doi.org/10.1002/aenm.202102389>
15. X. Shi, X. Dong, Y. He, P. Yan, S. Zhang et al., Photoswitchable chlorine vacancies in ultrathin Bi_4_O_5_Cl_2_ for selective CO_2_ photoreduction. ACS Catal. **12**(7), 3965-3973 (2022). <https://doi.org/10.1021/acscatal.2c00157>
16. J. Wu, X. Li, W. Shi, P. Ling, Y. Sun et al., Efficient visible‐light‐driven CO_2_ reduction mediated by defect‐engineered BiOBr atomic layers. Angew. Chem. Int. Ed. **130**(28), 8855-8859 (2018). https://doi.org/10.1002/anie.201803514
17. J. Di, C. Zhu, M. Ji, M. Duan, R. Long et al., Defect‐rich Bi1_2_O_17_Cl_2_ nanotubes self‐accelerating charge separation for boosting photocatalytic CO_2_ reduction. Angew. Chem. Int. Ed. **57**(45), 14847-14851 (2018). https://doi.org/10.1002/anie.201809492
18. X. Shi, X. Dong, Y. He, P. Yan, F. Dong, Light-induced halogen defects as dynamic active sites for CO_2_ photoreduction to CO with 100% selectivity. Sci. Bull. **67**(11), 1137-1144 (2022). <https://doi.org/10.1016/j.scib.2022.01.013>
19. L. Hao, L. Kang, H. Huang, L. Ye, K. Han et al., Surface‐halogenation‐induced atomic‐site activation and local charge separation for superb CO_2_ photoreduction. Adv. Mater. **31**(25), 1900546 (2019). <https://doi.org/10.1002/adma.201900546>
20. J. Di, C. Chen, C. Zhu, P. Song, J. Xiong et al., Bismuth vacancy-tuned bismuth oxybromide ultrathin nanosheets toward photocatalytic CO_2_ reduction. ACS Appl. Mater. Interfaces **11**(34), 30786-30792 (2019). <https://doi.org/10.1021/acsami.9b08109>
21. L. Wang, R. Wang, T. Qiu, L. Yang, Q. Han et al., Bismuth vacancy-induced efficient CO_2_ photoreduction in BiOCl directly from natural air: a progressive step toward photosynthesis in nature. Nano Lett. **21**(24), 10260-10266 (2021). <https://doi.org/10.1021/acs.nanolett.1c03249>
22. Y. Chen, F. Wang, Y. Cao, F. Zhang, Y. Zou et al., Interfacial oxygen vacancy engineered two-dimensional g-C_3_N_4_/BiOCl heterostructures with boosted photocatalytic conversion of CO_2_. ACS Appl. Energy Mater. **3**(5), 4610-4618 (2020). <https://doi.org/10.1021/acsaem.0c00273>
23. G. Liu, B. Wang, X. Zhu, P. Ding, J. Zhao et al., Edge‐site‐rich ordered macroporous BiOCl triggers C=O activation for efficient CO_2_ photoreduction. Small **18**(6), 2105228 (2021). <https://doi.org/10.1002/smll.202105228>
24. Y. Shi, G. Zhan, H. Li, X. Wang, X. Liu et al., Simultaneous manipulation of bulk excitons and surface defects for ultrastable and highly selective CO_2_ photoreduction. Adv. Mater. **33**(38), 2100143 (2021). https://doi.org/10.1002/adma. [C](https://doi.org/10.1039/D0NR08314C)202100143
25. Y. Li, Y. Liu, H. Mu, R. Liu, Y. Hao et al., The simultaneous adsorption, activation and in situ reduction of carbon dioxide over Au-loading BiOCl with rich oxygen vacancies. Nanoscale **13**(4), 2585-2592 (2021). <https://doi.org/10.1039/D0NR08314C>
26. Y. Shi, H. Shou, H. Li, G. Zhan, X. Liu et al., Visible light-driven conversion of carbon-sequestrated seawater into stoichiometric CO and HClO with nitrogen-doped BiOCl atomic layers. Angew. Chem. Int. Ed. **62**(24), e202302286 (2023). <https://doi.org/10.1002/anie.202302286>
